# Efficacy of comprehensive unit-based safety program to prevent ventilator associated-pneumonia for mechanically ventilated patients in China: A propensity-matched analysis

**DOI:** 10.3389/fpubh.2022.1029260

**Published:** 2022-12-15

**Authors:** Xiaomeng Yi, Xuxia Wei, Mi Zhou, Yingying Ma, Jinfeng Zhuo, Xin Sui, Yuling An, Haijin Lv, Yang Yang, Huimin Yi

**Affiliations:** ^1^Surgical Intensive Care Unit, The Third Affiliated Hospital of Sun Yat-sen University, Guangzhou, China; ^2^Transplantation Intensive Care Unit, The Third Affiliated Hospital of Sun Yat-sen University, Guangzhou, China; ^3^Department of Hepatic Surgery, The Third Affiliated Hospital of Sun Yat-sen University, Guangzhou, China

**Keywords:** comprehensive unit-based safety program, ventilator-associated pneumonia, propensity-matched analysis, ventilator-associated pneumonia bundle, safety culture

## Abstract

**Background:**

Ventilator-associated pneumonia (VAP) is the most common healthcare-associated infection (HAI) in patients with mechanical ventilation. VAP is largely preventable, and a comprehensive unit-based safety program (CUSP) has effectively reduced HAI. In this study, we aim to comprehensively investigate the effect of implementing the CUSP in patients requiring mechanical ventilation.

**Methods:**

In this uncontrolled before-and-after trial conducted in two intensive care unit (ICU) settings in China, patients requiring invasive mechanical ventilation were enrolled. Patients were divided into two groups based on the implementation of CUSP. The primary outcome was the incidence of VAP. The secondary outcomes were the time from intubation to VAP, days of antibiotic use for VAP treatments, rate of other infection, length of stay (LOS) in ICU, hospital LOS, and safety culture score. Joinpoint regression analysis was used to test the changes in trends of VAP rate for statistical significance. Propensity score matching (1:1 matching) was used to reduce the potential bias between CUSP and no CUSP groups. Univariate and multivariate logistic/linear regression analyses were performed to evaluate the association between the use of CUSP and clinical outcomes. This study was registered at the Chinese Clinical Trial Registry (chictr.org.cn), registration number: ChiCTR1900025391.

**Results:**

A total of 1,004 patients from the transplant ICU (TICU) and 1,001 patients from the surgical ICU (SICU) were enrolled in the study from January 2016 to March 2022. Before propensity score matching, the incidences of VAP decreased from 35.1/1,000 ventilator days in the no CUSP group to 12.3/1,000 ventilator days in the CUSP group in the TICU setting (adjusted odds ratio [OR], 0.30; 95% confidence interval [CI], 0.15–0.59). The results of the joinpoint regression analysis confirmed that the implementation of CUSP significantly decreased the incidences of VAP. After propensity score matching in TICU setting, the CUSP group reported a lower incidence of VAP (30.4 vs. 9.7‰, *P* = 0.003; adjusted OR = 0.26, 95% CI: 0.10–0.76), lower wound infection (3.4 vs. 0.9%, *P* = 0.048; adjusted OR = 0.73, 95% CI: 0.50–0.95), shorter ICU LOS [3.5(2.3–5.3) vs. 2.5(2.0–4.5) days; *P* = 0.003, adjusted estimate = −0.34, 95% CI: −0.92 to −0.14], and higher safety culture score (149.40 ± 11.74 vs. 153.37 ± 9.74; *P* = 0.002). Similar results were also observed in the SICU setting between the no CUSP and CUSP group.

**Conclusions:**

The implementation of CSUP for patients receiving mechanical ventilation could significantly reduce the incidences of VAP, and other infections, prolong the time until the VAP occurrence, reduces the days of antibiotic use for VAP, shorten the ICU and hospital LOS, and enhance the awareness of safety culture.

## Introduction

Ventilator-associated pneumonia (VAP) in patients requiring mechanical ventilation is the most frequently encountered healthcare-associated infection (HAI) in intensive care unit (ICU) settings (1). VAP affects 5–40% of patients receiving invasive mechanical ventilation for longer than 48 h; however, the number of incidences differs due to criteria used to identify VAP, type of ICU setting, and the country (2). The incidences of VAP in hospitals in North America and Europe are 1–2.5 cases/1,000 and 18.3/1,000 ventilator-days, respectively (3, 4). However, the incidences of VAP in Chinese hospitals are relatively high. For example, Chinese Society of Critical Care Medicine guidelines have reported 8.4–49.3 VAP episodes per 1,000 ventilator days (5). Additionally, VAP is associated with higher rates of morbidity and mortality in patients, more extended hospital stays, and higher medical expenses. Given that VAP is mostly preventable, strategies aimed to prevent/reduce the frequency of this infection are a major challenge for healthcare professionals.

Currently, evidence-based guidelines indicate that the prevention of VAP is feasible by implementing specific interventions together simultaneously. This tactic is called “a VAP bundle” (6, 7). The VAP bundle was used to reduce the incidences of VAP incidence and has become the focus of multiple international organizations (7–9). In recent years, reports suggest the effect of the VAP bundle in VAP prevention has had negative results (10, 11), which was attributed to the relatively poor medical practices for evidence-based strategies and safety culture. Studies show that to reduce the incidences of VAP, correct and efficient use of the VAP bundle was required. Further, regularly evaluating the medical and nursing staff and sustainable improvements are recommended to increase long-term compliance and execution (12).

Using a validated and structured framework, the comprehensive unit-based safety program (CUSP) was designed to improve the cooperation and safety culture to help organizations learn from previous mistakes (13). The CUSP was developed by the patient safety research team at the Johns Hopkins Hospital, Baltimore, Maryland (14). CUSP is a repetitive process that instructs multidisciplinary teams on the science of safety. It requires them to identify and learn from the flaws, implement improvement strategies, and form partnerships with senior leaders. The implementation of CUSP has achieved great success in lowering the rate of central line-associated bloodstream infection (CLABSI) rates (15), nosocomial infection (16), surgical complications (17), medication errors, and associated costs (18) in developed nations of Europe and North America. However, there are no reports in China regarding the use of CUSP in VAP prophylaxis in patients requiring mechanical ventilation.

To successfully implement CUSP, since 2014, our team has partnered with the Armstrong Center for Patient Safety and Quality (AIPSQ) at Johns Hopkins University, where CUSP was conceptualized and pioneered. We hypothesized that using CUSP could reduce the problems associated with implementing the VAP bundle and resolve factors interfering with the use of the VAP bundle. This will help reduce the incidence of VAP, shorten the length of ICU and hospital stay, and improve safety culture awareness.

## Methods

### Setting and organization of the ICUs

Patients admitted to the transplantation ICU (TICU) and surgical ICU (SICU) of the Third Affiliated Hospital of Sun Yat-sen University between January 2016 and March/December 2020 were enrolled for this clinical trial. The TICU had a mixed population of patients undergoing liver and renal transplantation. The SICU had a mixed set of patients with neurologic concerns, trauma, and surgical patients. The CUSP was fully implemented in April 2019. The patients were categorized into two groups based on the implementation of CUSP (no CUSP group vs. CUSP group).

The inclusion criteria of the patients were as follows: (1) patients in TICU/SICU requiring invasive mechanical ventilation for more than 24 h; (2) patients with a Child-Pugh score >7 or Acute Physiology and Chronic Health Evaluation (APACHE) II score >9.

The exclusion criteria of the patients were as follows: (1) patients died within 48 h of admission; (2) patients ventilated with a tracheotomy; (3) mechanical ventilation more than 72 h prior to enrollment; (4) patients with VAP diagnosed prior to enrollment.

All procedures performed in studies involving human participants were in accordance with the ethical standards of the institutional and/or national research committee and with the 1964 Helsinki Declaration and its later amendments or comparable ethical standards. The study was reviewed and approved by the Ethics Committee of the Third Affiliated Hospital of Sun Yat-sen University. Due to the retrospective nature of the study, the need for informed consent was waived. This study was registered at the Chinese Clinical Trial Registry (chictr.org.cn), registration number: ChiCTR1900025391.

### Team formation

A multidisciplinary CUSP team was established in August 2014 to implement evidence-based practice for patients receiving mechanical ventilation and VAP. The team was led by an intensivist and included respiratory therapists, nurses, other physicians, quality management personnel, and infection control practitioners.

### Definition and diagnosis of VAP

VAP is an infection of the pulmonary parenchyma in patients requiring invasive mechanical ventilation for more than 48 h. The diagnosis of VAP requires clinical suspicion (19) (≥two criteria including leukocytosis of >10,000 cells/mL or leukopenia of < 4,000 cells/mL, fever of >38.5°C, a new or persistent infiltrate on chest radiography and purulent tracheobronchial secretions) and confirmation by the positive quantitative cultures of distal pulmonary sampling like plugged telescoping catheter (significant threshold ≥10^3^ colony-forming units (CFU) /mL) or broncho-alveolar lavage fluid (significant threshold ≥10^4^ CFU/mL) or quantitative endotracheal aspirate pulmonary secretion samples (significant threshold ≥10^6^ CFU/mL), according to the international guidelines.

The primary route of VAP is through micro inhalation of microorganisms which have colonized the oropharyngeal tract. The microorganisms involved in VAP can be widely varied. Common pathogens include aerobic gram-negative bacilli (e.g., *Escherichia coli, Klebsiella pneumoniae, Enterobacter* spp., *Pseudomonas aeruginosa, Acinetobacter* spp.), gram-positive cocci (e.g., *Staphylococcus aureus*, including *methicillin-resistant S. aureus* [MRSA], *Streptococcus* spp.) and fungus (e.g., *Candidiasis, Aspergillosis*).

### Regulation for ventilator circuit changes

For immunocompromised patients in TICU, ventilator circuits with dual heating (Evaqua 2, *F&P*) were changed once a week. For immunocompetent patients, circuit changes were ordered every 2 weeks.

### CUSP development and intervention

The CUSP was designed to enhance teamwork, communication, and patient safety culture, thereby implementing the VAP bundle into effective practices.

**Step1:** Summarize the existing research evidence.

a. Identifying manipulable factors to reduce the occurrence of VAP.
b. Selecting factors combined with the VAP bundle to maximize the benefit and minimizes hurdles during the implementation of the VAP bundle.

By conducting a literature review and in-depth communication with the team, a VAP bundle was constructed, including (1) Elevating the head of the bed 30–45 degrees; (2) Daily wake-up and weaning assessments; (3) Avoiding the overuse of anti-acid prophylaxis: Daily screen the risk factors of stress ulcer. We recommend stopping anti-acid prophylaxis, when the risk of gastrointestinal is bleeding lower than 4%; (4) Oral care with 0.12% chlorhexidine rinse twice a day; (5) Subglottic secretion suction: If patients anticipated to need MV for >24 h, we intubated with a TaperGuard evacuation oral tracheal tube (Covidien, Mans-field, MA). The subglottic ports were irrigated every 6 h with 10 ml of distilled sterile water according to the manufacturer's recommendation; and (6) Dual hand hygiene: The hands of medical staff are an important means of transmitting the VAP pathogen. The seven-step hand washing procedure should be strictly followed before and after any medical procedure.

**Step2:** Identifying obstacles that prevent VAP bundle implementation (monthly summarized).

a. Observing the medical staff's compliance to the VAP bundle.
b. The “full track” approach identifies flaws at each step of execution;
c. Taking note of all concerns of the medical staff and identifying the potential pros and cons in the implementation process.

**Step3:** Evaluation of the performance (monthly summarized).

a. Creating an assessment scale based on the specifications;
b. Revising and testing the applicability of the scale;
c. The scale was used for the initial baseline assessment.

**Step4:** Ensuring eligible patients receive the standard of care.

CUSP was implemented on patients requiring invasive mechanical ventilation and should follow the “4E” (Engage, Educate, Execute, Evaluate) principle.

Engage: The CUSP leader informs the team members about the importance of improving the implementation of the VAP bundle to reduce VAP and encourages them to participate in improving their work.

Educate: The CUSP leader educates the team members on VAP bundle implementation and associated details. They also document the problems related to the implementation of the VAP bundle.

Execute: The CUSP team developed a “toolbox” to improve VAP bundle procedures to overcome defects and obstacles, including creating standardized procedures, improving the workflow, creating reminders, and learning from previous mistakes.

Evaluate: The CUSP team periodically evaluated the implementation and effects of the VAP bundle.

### Communications

The meeting with the CUSP team was conducted every month. Bi-monthly webinars were held between the CUSP team and AIPSQ from Johns Hopkins University.

### Data collection and outcome measure

To evaluate the effect of implementing CUSP, clinical information about the patients was obtained, and Hospital Survey on Patient Safety (HSPS) was conducted. For each patient in TICU, the following information like the age, sex, body mass index (BMI), weight, height, previous medical history, levels of procalcitonin (PCT), creatinine, albumin, total bilirubin (TBIL), a model for end-stage liver disease (MELD) score (indicating the severity of liver disease), international normalized ratio (INR), Child-Pugh score (range 0–15, where higher scores indicated more severe illness), hemodialysis (HD), pre-transplantation infection, HBsAg positive, priority transplant score, warm ischemia time (WIT), anhepatic phase, and the operation time at baseline were recorded. For patients admitted to SICU, the following information of the eligible patients like the patient's age, sex, APACHE II score (range 0–71, where higher scores indicated more severe illness), PCT, white blood cell (WBC) count, levels of alanine aminotransferase (ALT), albumin were extracted from medical records at baseline.

The primary endpoint of the study was the incidence of microbiologically confirmed VAP in patients intubated for ≥48 h. The microbiological test results of VAP patients were recorded. The secondary endpoints of the study were duration of mechanical ventilation, ventilator-free days (VFDs) at day 28, the time elapsed from the first diagnosis of VAP, reintubation, other nosocomial infection (pulmonary/wound /opportunistic/ bloodstream infection), the time elapsed until pulmonary/wound/opportunistic infection, pleura effusion, the time elapsed until pleura effusion, day of antibiotics use for VAP treatment, length of stay (LOS) in the ICU and the hospital, ICU, and hospital mortality.

HSPS was conducted at the start and the end of the clinical trial. The safety culture items consisted of 12 dimensions and two individual entries. The questionnaire provided is shown in Supplementary material 1.

### Statistical analysis

The normality of data was determined using the Kolmogorov-Smirnov test. The parametric data were analyzed using the student's *t*-test and represented as the mean (standard deviation). The non-parametric data were analyzed using the Mann–Whitney test and represented as median [interquartile range (IQR): 25th−75th percentiles]. Categorical variables were analyzed by the Chi-squared or Fisher exact test, and the data was represented as a number (%).

VAP incidence was reported as the incidence per 1,000 ventilator days and was calculated every quarter. Quarterly VAP incidence = quarter VAP events/quarter ventilator days. Total VAP incidence = total VAP events/total ventilator days.

Joinpoint regression analysis was used to identify significant shifts and trends in VAP incidences. The most significant joinpoint counts were used in the final model, and a quarter percentage change (QPC) was computed for each slope. Joinpoint Regression Program version 4.9.0.0 (Statistical Applications and Research Branch of the National Cancer Institute; https://surveillance.cancer.gov/joinpoint/) was used for performing Joinpoint regression analysis. The cumulative incidence of VAP was evaluated by considering the competing risk of ICU discharge (discharged alive or dead).

To reduce the differences in baseline characteristics of the patient between no CUSP and CUSP groups, the propensity-matched analysis was performed. 1:1 matching by propensity score was performed using logistic regression. A caliper width equal to 0.02 standard deviation was used to account for baseline covariates potentially associated with CUSP implementation.

The effect of CUSP on outcomes (categorical variables) was assessed using logistic regression models and expressed as a crude/adjusted Odds ratio (OR) with 95% confidence intervals (CI). CUSP effects on outcomes (continuous variables) were evaluated using linear regression models and reported as crude/adjusted estimated regression coefficient with 95% CI.

*Post-hoc* sensitivity analysis was conducted on patients requiring mechanical ventilation for at least 48–72 h.

SPSS Statistics V 22.0 and R package software (version 4.0.4) were used to perform statistical analysis. Except for cases where a specific *P*-value was specified, a *P* < 0.05 (two-sided) was considered statistically significant.

## Results

### Study population

Between January 2016 to March 2022, a total of 1,004 patients received mechanical ventilation in TICU, of which 562 patients in the no CUSP group and 442 patients in the CUSP group met the inclusion criteria. The demographic and baseline characteristics are listed in Table 1. No differences in most of the clinical characteristics at admission were observed in patients in the no CUSP and CUSP groups. However, differences in spontaneous bacterial peritonitis, esophagogastric fundus vein bleeding (EGVB), PCT, HD, pre-transplant infections, priority transplant score, WIT, anhepatic phase, and operation time was observed in patients in both the groups.

**Table 1:** Baseline characteristics of no CUSP group and CUSP group in the TICU setting.

**Variables**	**Before propensity matching**			**After propensity matching**		
	**No CUSP (*n* = 562)**	**CUSP (*n* = 442)**	***P*-value**	**No CUSP (*n* = 238)**	**CUSP (*n* = 238)**	***P*-value**
Male sex, No. (%)	487 (87.5)	383 (85.5)	0.349	203 (85.3)	205 (86.1)	0.793
Age, median (IQR), year	49 (42–56)	50 (42–56)	0.794	49 (41–56)	50 (43–55)	0.821
Weight, mean (SD), kg	65.1 (11.0)	65.7 (11.3)	0.093	65.8 (11.2)	65.6 (11.0)	0.560
Height, mean (SD), cm	168.2 (6.1)	167.8 (6.3)	0.518	168.0 (6.4)	168.2 (6.3)	0.777
BMI, mean (SD)	23.0 (3.2)	23.2 (3.2)	0.526	23.2 (3.2)	23.1 (3.1)	0.156
**Previous medical history, No. (%)**						
Hypertension	70 (12.5)	56 (12.7)	0.927	29 (12.2)	24 (10.1)	0.466
Diabetes mellitus	89 (15.8)	70 (15.8)	1.000	42 (17.6)	33 (13.9)	0.258
Cancer	231 (41.1)	193 (43.7)	0.415	92 (38.7)	98 (41.2)	0.574
Progressive hyperbilirubinemia	463 (82.4)	361 (81.7)	0.771	199 (83.6)	198 (83.2)	0.902
Refractory ascites	437 (77.8)	353 (79.9)	0.419	185 (77.7)	187 (78.6)	0.824
Spontaneous bacterial peritonitis	39 (7.0)	51 (11.5)	0.012*	18 (7.6)	23 (9.7)	0.414
Hepatic encephalopathy	166 (29.5)	122 (27.6)	0.501	67 (28.2)	61 (25.6)	0.535
EGVB	14 (2.5)	2 (0.5)	0.010*	2 (0.8)	2 (0.8)	1.000
HRS	108 (19.2)	96 (21.7)	0.328	49 (20.6)	45 (18.9)	0.645
PCT, median (IQR), ng/mL	6.8 (2.4–20.6)	3.1 (1.3–8.4)	0.001*	5.8 (1.8–13.0)	3.8 (1.6–12.1)	0.293
Creatinine, median (IQR), umol/L	74.5 (60.8–97.0)	72.0 (58.5–89.0)	0.123	74 (58–97)	72 (56.8–94.5)	0.737
Albumin, median (IQR), g/L	35.2 (32.1–38.8)	35.1 (31.8–38.8)	0.248	35.1 (32.0–38.9)	35.6 (31.9–39.7)	0.640
TBIL, median (IQR), umol/L	153.9 (30.3–454.2)	145.9 (25.6–422.2)	0.123	174.4 (31.7–434.5)	147.0 (26.7–426.9)	0.462
INR, median (IQR)	1.9 (1.3–3.3)	1.9 (1.3–3.2)	0.680	2.0 (1.3–3.1)	2.0 (1.3–3.3)	0.771
MELD score, mean (SD)	24.3 (12.0)	24.1 (12.6)	0.071	24.8 (11.9)	24.0 (12.1)	0.641
Child-Pugh score, mean (SD)	9.8 (2.6)	9.9 (2.6)	0.237	10.0 (2.5)	9.8 (2.5)	0.433
HD, No. (%)	80 (14.3)	100 (22.6)	0.001*	46 (19.3)	36 (15.1)	0.225
Pretransplant infection, No. (%)	228 (40.6)	247 (55.9)	0.001*	122 (51.3)	114 (47.9)	0.463
HBsAg positive, No. (%)	448 (79.7)	346 (78.3)	0.579	190 (79.8)	186 (78.2)	0.653
Priority transplant score, mean (SD)	3.4 (1.2)	3.8 (1.1)	0.010*	3.6 (1.2)	3.6 (1.0)	0.127
WIT, median (IQR), min	0 (0–10)	0 (0–0)	0.001*	0 (0–5)	0 (0–0)	0.132
Anhepatic phase, median (IQR), min	46 (40–54)	45 (38–53)	0.019*	45 (40–54)	45 (38–53)	0.391
Operation time, median (IQR), h	7 (6–8)	6 (5–7)	0.001*	7 (6–7)	6 (6–7)	0.167

CUSP, comprehensive unit-based safety program; TICU, transplantation intensive care medicine; BMI, body mass index; IQR, interquartile range; SD, standard deviation; EGVB, esophagogastric fundus vein bleeding; HRS, hepatorenal syndrome; PCT, procalcitonin; TBIL, total bilirubin; INR, international normalized ratio; MELD, model for end-stage liver disease; HD, hematodialysis; WIT, warm ischemia time. ^*^P < 0.05.

Between January 2016 and December 2020, a total of 1,001 patients were admitted to in SICU, of which 613 patients were in the no CUSP group and 388 patients in the CUSP group were enrolled for the study. The demographic and baseline characteristics are summarized in Table 2. A significant difference in the sex of the patients between both groups was observed at admission.

**Table 2:** Baseline characteristics of no CUSP group and CUSP group in the SICU setting.

**Variables**	**Before propensity matching**			**After propensity matching**		
	**No CUSP (*n* = 613)**	**CUSP (*n* = 388)**	***P*-value**	**No CUSP (*n* = 198)**	**CUSP (*n* = 198)**	***P*-value**
Male sex, No. (%)	388 (63.3)	270 (69.6)	0.041*	121 (61.1)	127 (64.1)	0.533
Age, median (IQR), year	54 (41–66)	55 (44–66)	0.569	61 (49–71)	59 (49.8–71)	0.904
APACHE II score, mean (SD)	18.7 (4.3)	16.6 (3.9)	0.086	17.4 (4.1)	17.3 (4.0)	0.260
PCT, median (IQR), ng/mL	0.92 (0.25–4.86)	0.61 (0.22–2.72)	0.121	0.65 (0.20–2.27)	0.54 (0.18–2.22)	0.602
ALT, median (IQR), U/L	18.0 (12.0–33.0)	19.0 (11.0–33.5)	0.746	17.0 (12.0–33.2)	17.0 (11.0–29.0)	0.182
Albumin, median (IQR), g/L	32.2 (27.6–36.0)	31.7 (28.3–36.4)	0.597	32.6 (28.4–36.2)	31.5 (28.5–36.0)	0.437
WBC count, median (IQR), 10^9^/L	11.38 (8.22–14.65)	11.15 (7.56–14.13)	0.203	11.60 (8.22–14.44)	11.00 (7.53–13.88)	0.100

CUSP, comprehensive unit-based safety program; SICU, surgical intensive care medicine; APACHE II, acute physiology and chronic health evaluation II; IQR, interquartile range; SD, standard deviation; PCT, procalcitonin; ALT, alanine aminotransferase; WBC, white blood cell. ^*^P < 0.05.

### Incidence and clinical features of VAP

**Figure 1:**
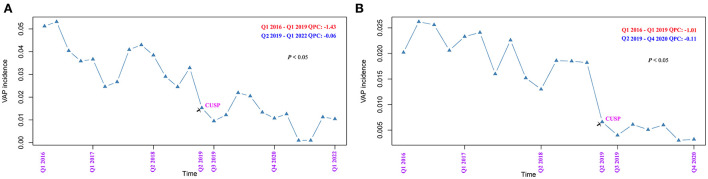
Joinpoint regression analysis of the occurrence of ventilator-associated pneumonia (VAP) in TICU and SCIU settings by quarter. **(A)** TICU. **(B)** SICU. The asterisks indicate a statistical significance, *P*-value (*P < 0.05; **P < 0.01; ***P < 0.001). TICU, transplantation intensive care unit; SICU, surgical intensive care unit; APC, annual percentage change.

**Figure 2:**
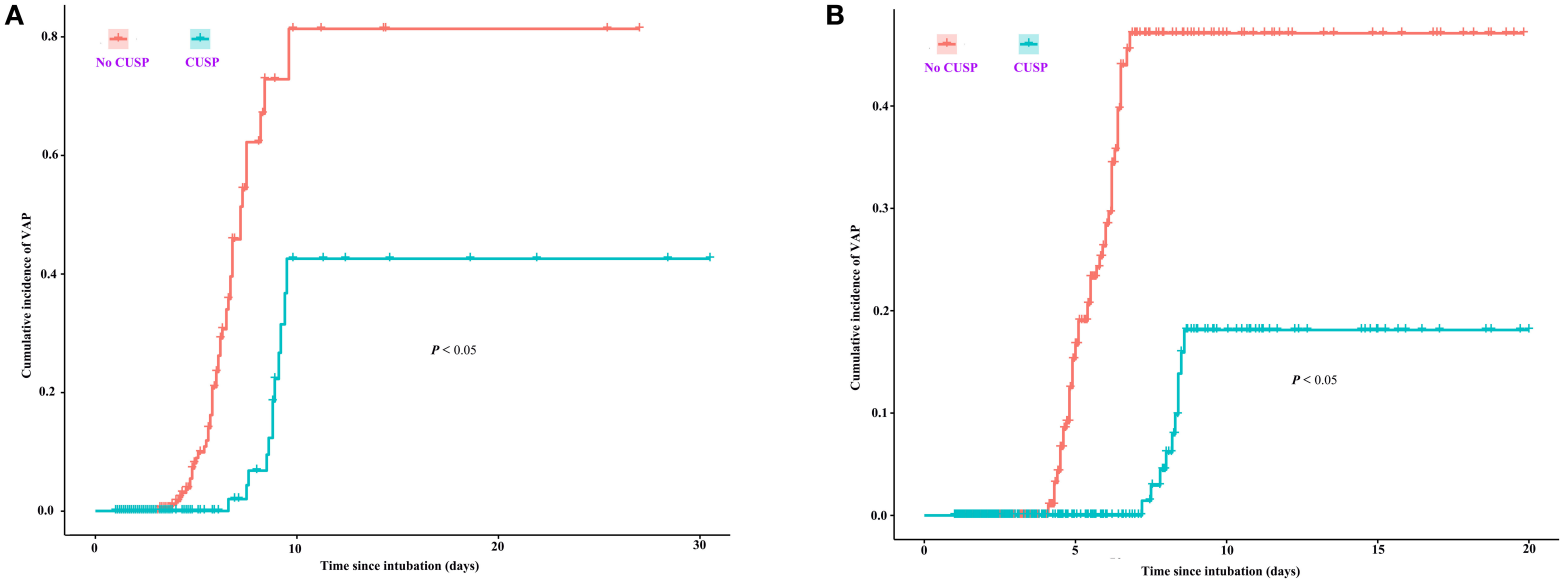
Cumulative proportion curves showed that the VAP incidence in the no CUSP group was continuously distributed at the significantly higher side compared to the CUSP group in both TICU **(A)** and SICU **(B)** settings. The asterisks indicate a statistical significance, *P*-value (*P < 0.05; **P < 0.01; ***P < 0.001). TICU, transplantation intensive care unit; SICU, surgical intensive care unit.

The quarterly VAP incidence in TICU ranged from 53.1 to 1.0 VAP/1,000 ventilator days, and SICU ranged from 26.2 to 3.0 VAP/1,000 ventilator days, as shown in Figures 1A,B, respectively. Joinpoint regression analysis (Figure 1) revealed that Quarter (Q)2 of 2019 (CUSP implementation) was the most significant joinpoints in TICU (Q1 of 2016–Q1 of 2019 of QPC: −1.43 and Q2 of 2019–Q1 of 2022 QPC: −0.06). The most significant joinpoints in SICU were Q1 of 2016–Q1 of 2019 QPC: −1.01; Q2 of 2019–Q4 of 2020 QPC: −0.11. The cumulative proportion curves showed that VAP incidence in patients in the no CUSP group was continuously distributed at the significantly higher side compared to patients in the CUSP group in both TICU (Figure 2A) and SICU (Figure 2B) settings after adjustment for the competing risks of discharged alive and dead.

The microbiological testing results of VAP are shown in Supplementary Table S1 (TICU) and Supplementary Table S2 (SICU). In the TICU setting, *Acinetobacter baumannii* (43.5%), *Klebsiella pneumoniae* (15.2%), and *Etenotrophomonas maltophilia* (15.2%) were the most common causative microorganisms in patients in the no CUSP group. Similar results were observed in patients in the CUSP group. The incidence of multidrug resistant (MDR) pathogens was 52.2 and 50% in the no CUSP and CUSP groups, respectively. In the SICU setting, *Acinetobacter baumannii* was the most common causative microorganisms, followed by *Pseudomonas aeruginosa* in both no CUSP and CUSP groups. The incidence of multidrug resistant (MDR) pathogens was 55.6 and 50% in the no CUSP and CUSP groups, respectively.

### Immunomodulating therapies and parenteral nutrition

In the TICU setting, the number of immunomodulatory treatments was 534 (95.0%) and 425 (96.2%) in the no CUSP and CUSP groups, respectively. The number of parenteral nutrition in the no CUSP and CUSP groups was 523 (93.1%) and 407 (92.1%), respectively. There was no significant difference between the two groups. In the SICU setting, the number of immunomodulating therapies was 74 (12.1%) and 45 (11.6%) in the no CUSP and CUSP groups, respectively. The number of parenteral nutrition in the no CUSP and the CUSP groups was 521 (85.0%) and 349 (83.8%), respectively. There was no significant difference between the two groups.

### Propensity-matched analysis

**Figure 3:**
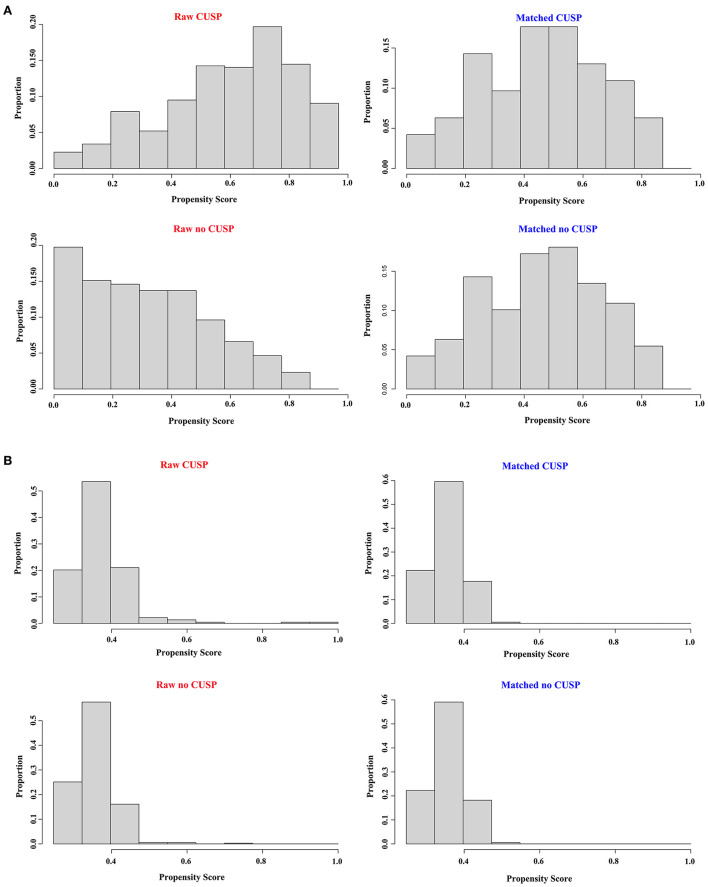
Distribution of propensity scores in the TICU and SCIU setting between the no CUSP and CUSP groups. **(A)** Before matching [left panel **(A)**] and after matching [right panel **(A)**] in the TICU setting. **(B)** Before matching [left panel **(B)**] and after matching [right panel **(B)**] in the SICU setting. TICU, transplantation intensive care unit; SICU, surgical intensive care unit.

As described in our previous results, there were significant differences between the two groups in baseline data in both the TICU and SICU settings. The propensity-matched analysis was performed to reduce the inter-group imbalances in the baseline data in both the TICU and SICU settings (Figures 3A,B). The results of propensity score matching generated 238 pairs of patients in the TICU setting and 198 pairs of patients in SICU settings between the two groups. However, no significant differences were observed between the two groups (Tables 1, 2).

### Primary endpoint

Before the propensity score matching in TICU, the total VAP incidences were 35.1 VAP/1,000 ventilator days in the no CUSP and 12.3 VAP/1,000 ventilator days in the CUSP group (*P* = 0.001, Table 3). Multivariate logistic regression analysis revealed that the CUSP intervention had a lower rate of VAP incidence (adjusted odds ratio [OR], 0.30; 95% confidence interval [CI], 0.15–0.59; *P* = 0.003, Table 4). After propensity score matching in TICU, the total VAP rate was significantly higher in patients in the no CUSP group (30.4‰) than in the CUSP group (9.7‰) (*P* = 0.003; Table 3). Multivariate logistic regression revealed that CUSP was associated with a significant decrease of 74% (adjusted OR, 0.26; 95% CI, 0.10–0.76; *P* = 0.014) in VAP incidences in patients compared to patients who did not receive CUSP intervention (Table 4).

**Table 3:** Primary and secondary outcomes, based on the study group in the TICU setting.

**Variables**	**Before propensity matching**			**After propensity matching**		
	**No CUSP**	**CUSP**	***P*-value**	**No CUSP**	**CUSP**	***P*-value**
	**(*n* = 562)**	**(*n* = 442)**		**(*n* = 238)**	**(*n* = 238)**	
**Primary outcome**
VAP (per 1,000 ventilator-days), No. (‰)	46 (35.1)	12 (12.3)	0.001*	17 (30.4)	5 (9.7)	0.003*
**Secondary outcomes**
Days of mechanical ventilation median (IQR), days	3.6 (1.3–4.3)	3.4 (1.1–3.9)	0.001*	3.5 (1.3–4.3)	3.3 (1.0–3.6)	0.001*
Total ventilator days, days	1,310.3	974.1		559.8	506.8	
VFDs at day 28, median (IQR),days	24.4 (23.7–26.6)	26.6 (26.1–26.9)	0.001*	24.5 (23.7–26.7)	24.7 (24.3–26.9)	0.001*
Time until VAP, mean (SD), days	5.9 (1.2)	8.5 (0.8)	0.025*	5.9 (1.1)	8.6 (1.1)	0.003*
Reintubation, No. (%)	31 (5.6)	18 (4.2)	0.302	15 (6.4)	11 (4.7)	0.433
Pulmonary infection, No. (%)	120 (21.4)	67 (15.2)	0.061	50 (21.0)	40 (16.8)	0.067
Pleura effusion, No. (%)	232 (41.3)	133 (30.1)	0.076	98 (41.2)	81 (34.0)	0.075
Time until pleura effusion, median (IQR), days	1 (0–5)	1 (0–1)	0.001*	1 (0–5)	1 (0–2)	0.126
Wound infection, No. (%)	18 (3.2)	5 (1.1)	0.031*	8 (3.4)	2 (0.9)	0.048*
Opportunistic infection, No. (%)	129 (22.9)	83 (18.8)	0.109	53 (22.6)	45 (18.9)	0.108
Time until opportunistic infection, median (IQR), days	2.5 (0–9.3)	0 (0–5)	0.008*	0 (0–6.5)	0 (0–7)	0.683
Days of antibiotic use for VAP, mean (SD), days	18.3 (2.6)	14.3 (1.9)	0.041*	18.4 (3.2)	14.4 (2.3)	0.013*
ICU LOS, median (IQR), days	5.5 (4.3–6.4)	4.9 (4.0–6.8)	0.011*	5.5 (4.3–7.3)	4.5 (4.0–6.5)	0.012*
Hospital LOS, median (IQR), days	24 (18–32)	21 (16–27)	0.001*	24 (17–31)	20 (16–25)	0.001*
ICU mortality, No. (%)	41 (7.3)	27 (6.1)	0.458	17 (7.1)	15 (6.3)	0.714
Hospital mortality, No. (%)	66 (11.7)	49 (11.1)	0.745	32 (13.4)	28 (11.8)	0.581

TICU, transplantation intensive care medicine; VFDs, ventilator-free days; ICU, intensive care medicine; VAP, ventilator associated-pneumonia; IQR, interquartile range; SD, standard deviation; LOS, length of stay. ^*^P < 0.05.

**Table 4:** Logistic regression or linear regression analysis for outcomes based on the initiation of CUSP in the TICU setting.

**Variables**	**Before propensity matching**			**After propensity matching**		
	**No CUSP (*n* = 562)**	**CUSP (*n* = 442)**	***P*-value**	**No CUSP (*n* = 238)**	**CUSP (*n* = 238)**	***P*-value**
VAP (per 1,000 ventilator-days), No. (‰)	46 (35.1)	12 (12.3)		17 (30.4)	5 (9.7)	
Crude OR (95% CI)	1.00 (Ref.)	0.31 (0.16 to 0.60)	0.001*	1.00 (Ref.)	0.28 (0.10 to 0.77)	0.013*
Multivariate-adjusted OR (95% CI)	1.00 (Ref.)	0.30 (0.15 to 0.59)	0.003*	1.00 (Ref.)	0.26 (0.10 to 0.76)	0.014*
Days of mechanical ventilation median (IQR), days	3.6 (1.3–4.3)	3.4 (1.1–3.9)		3.5 (1.3–4.3)	3.3 (1.0–3.6)	
Crude estimate (95% CI)	0 (Ref.)	−0.25 (−0.60 to +0.07)	0.098	0 (Ref.)	−0.23 (−0.73 to +0.29)	0.386
Multivariate-adjusted estimate (95% CI)	0 (Ref.)	−0.24 (−0.56 to +0.08)	0.134	0 (Ref.)	−0.22 (−0.72 to +0.28)	0.405
VFDs at day 28, median (IQR), days	24.4 (23.7–26.6)	26.6 (26.1–26.9)		24.5 (23.7–26.7)	24.7 (24.3–26.9)	
Crude estimate (95% CI)	0 (Ref.)	0.42 (−0.30 to +0.74)	0.243	0 (Ref.)	0.23 (−0.27 to +0.71)	0.380
Multivariate-adjusted estimate (95% CI)	0 (Ref.)	0.38 (−0.71 to +0.81)	0.820	0 (Ref.)	0.22 (−0.28 to +0.70)	0.395
Time until VAP, mean (SD), days	5.9 (1.2)	8.5 (0.8)		5.9 (1.1)	8.6 (1.1)	
Crude estimate (95% CI)	0 (Ref.)	+2.50 (+1.94 to +3.23)	0.001*	0 (Ref.)	+2.63 (+1.43 to +3.82)	0.001*
Multivariate-adjusted estimate (95% CI)	0 (Ref.)	+2.29 (+1.81 to +3.37)	0.008*	0 (Ref.)	+2.33 (+1.25 to +3.99)	0.003*
Wound infection, No. (%)	18 (3.2)	5 (1.1)		8 (3.4)	2 (0.9)	
Crude OR (95% CI)	1.00 (Ref.)	0.35 (0.13 to 0.95)	0.039*	1.00 (Ref.)	0.77 (0.51 to 0.96)	0.040*
Multivariate-adjusted OR (95% CI)	1.00 (Ref.)	0.37 (0.13 to 0.94)	0.045*	1.00 (Ref.)	0.73 (0.50 to 0.95)	0.042*
Days of antibiotic use for VAP, mean (SD), days	18.3 (2.6)	14.3 (1.9)		18.4 (3.2)	14.4 (2.3)	
Crude estimate (95% CI)	0 (Ref.)	−3.30 (−5.38 to −2.25)	0.001*	0 (Ref.)	−3.99 (−6.92 to −1.08)	0.013*
Multivariate-adjusted estimate (95% CI)	0 (Ref.)	−3.99 (−5.61 to −2.51)	0.007*	0 (Ref.)	−3.99 (−7.20 to −0.08)	0.017*
ICU LOS, median (IQR), days	5.5 (4.3–6.4)	4.9 (4.0–6.8)		5.5 (4.3–7.3)	4.5 (4.0–6.5)	
Crude estimate (95% CI)	0 (Ref.)	−0.39 (−0.68 to −0.16)	0.028*	0 (Ref.)	−0.32 (−0.89 to −0.22)	0.036*
Multivariate-adjusted estimate (95% CI)	0 (Ref.)	−0.41 (−0.72 to −0.18)	0.032*	0 (Ref.)	−0.34 (−0.92 to −0.14)	0.038*
Hospital LOS, median (IQR), days	24 (18–32)	21 (16–27)		24 (17–31)	20 (16–25)	
Crude estimate (95% CI)	0 (Ref.)	−2.30 (−4.94 to −0.56)	0.012*	0 (Ref.)	−3.56 (−5.90 to −1.23)	0.003*
Multivariate-adjusted estimate (95% CI)	0 (Ref.)	−2.78 (−5.10 to −0.62)	0.014*	0 (Ref.)	−3.54 (−5.60 to −1.13)	0.003*

TICU, transplantation intensive care medicine; CUSP, comprehensive unit-based safety program; VFDs, ventilator-free days; ICU, intensive care medicine; VAP, ventilator associated-pneumonia; IQR, interquartile range; SD, standard deviation; LOS, length of stay; CI, confidence intervals; OR, odds ratio. ^*^P < 0.05.

Similarly, before and after propensity score matching in SICU, the total VAP rate was significantly lower in patients in the CUSP group compared to patients in the no CUSP group (Table 5). Further, the CUSP intervention significantly decreased the VAP incidences in patients (Table 6).

**Table 5:** Primary and secondary outcomes, based on the study group in the SICU setting.

**Variables**	**Before propensity matching**			**After propensity matching**		
	**No CUSP (*n* = 613)**	**CUSP (*n* = 388)**	***P*-value**	**No CUSP (*n* = 198)**	**CUSP (*n* = 198)**	***P*-value**
**Primary outcome**	
VAP (per 1,000 ventilator-days), No. (‰)	54 (19.1)	10 (4.8)	0.001*	16 (18.7)	6 (4.8)	0.004*
**Secondary outcomes**	
Days of mechanical ventilation median (IQR), days	2.6 (1.6–4.9)	2.7 (1.6–5.9)	0.232	2.8 (1.5–5.2)	3 (1.6–6.7)	0.146
Total ventilator days, days	2,822.6	2,095.5		854.2	1,262.9	
Time until VAP, mean (SD), days	5.3 (0.8)	8.1 (0.5)	0.008*	5.2 (0.6)	8.0 (0.5)	0.009*
Bloodstream infection, No. (%)	46 (7.5)	14 (3.6)	0.031*	20 (10.1)	6 (3.0)	0.005*
Days of antibiotic use for VAP, mean (SD), days	15.1 (1.6)	11.0 (1.4)	0.001*	15.3 (1.5)	10.7 (1.6)	0.008*
ICU LOS, median (IQR), days	4.0 (2.5–9.0)	2.5 (2.0–6.0)	0.005*	7 (3–12)	5 (2–9.25)	0.020*
Hospital LOS, median (IQR), days	25 (19–33)	20 (14–25)	0.001*	26 (16–33)	21 (14–24)	0.009*

SICU, surgical intensive care medicine; ICU, intensive care medicine; VAP, ventilator associated-pneumonia; IQR, interquartile range; SD, standard deviation; LOS, length of stay. ^*^P < 0.05.

**Table 6:** Logistic regression or linear regression analysis for outcomes based on the initiation of CUSP in the SICU setting.

**Variables**	**Before propensity matching**			**After propensity matching**		
	**No CUSP (*n* = 562)**	**CUSP (*n* = 442)**	***P*-value**	**No CUSP (*n* = 238)**	**CUSP (*n* = 238)**	***P*-value**
VAP (per 1,000 ventilator-days), No. (‰)	54 (19.1)	10 (4.8)		16 (18.7)	6 (4.8)	
Crude OR (95% CI)	1.00 (Ref.)	0.30 (0.15 to 0.60)	0.001*	1.00 (Ref.)	0.35 (0.14 to 0.93)	0.035*
Multivariate-adjusted OR (95% CI)	1.00 (Ref.)	0.36 (0.16 to 0.80)	0.001*	1.00 (Ref.)	0.36 (0.14 to 0.94)	0.038*
Time until VAP, mean (SD), days	5.3 (0.8)	8.1 (0.5)		5.2 (0.6)	8.0 (0.5)	
Crude estimate (95% CI)	0 (Ref.)	+2.78 (+2.26 to +3.30)	0.002*	0 (Ref.)	+2.80 (+2.21 to +3.38)	0.001*
Multivariate-adjusted estimate (95% CI)	0 (Ref.)	+2.78 (+2.40 to +3.17)	0.004*	0 (Ref.)	+2.80 (+2.25 to +3.35)	0.003*
Bloodstream infection, No. (%)	46 (7.5)	14 (3.6)		20 (10.1)	6 (3.0)	
Crude OR (95% CI)	1.00 (Ref.)	0.48 (0.31 to 0.89)	0.021*	1.00 (Ref.)	0.30 (0.21 to 0.93)	0.018*
Multivariate-adjusted OR (95% CI)	1.00 (Ref.)	0.54 (0.29 to 0.93)	0.035*	1.00 (Ref.)	0.32 (0.24 to 0.96)	0.020*
Days of antibiotic use for VAP, mean (SD), days	15.1 (1.6)	10.9 (1.4)		15.3 (1.5)	10.7 (1.6)	
Crude estimate (95% CI)	0 (Ref.)	−4.13 (−5.20 to −3.07)	0.002*	0 (Ref.)	−3.59 (−6.13 to −3.04)	0.011*
Multivariate-adjusted estimate (95% CI)	0 (Ref.)	−4.13 (−5.22 to −3.04)	0.005*	0 (Ref.)	−3.59 (−6.34 to −2.84)	0.015*
ICU LOS, median (IQR), days	4.0 (2.5–9.0)	2.5 (2.0–6.0)		7 (3–12)	5 (2–9.25)	
Crude estimate (95% CI)	0 (Ref.)	−1.44 (−1.88 to −0.25)	0.024*	0 (Ref.)	−1.95 (−2.58 to −1.28)	0.016*
Multivariate-adjusted estimate (95% CI)	0 (Ref.)	−1.38 (−182 to −0.22)	0.035*	0 (Ref.)	−1.95 (−2.34 to −1.04)	0.019*
Hospital LOS, median (IQR), days	25 (19–33)	20 (14–25)		26 (16–33)	21 (14–24)	
Crude estimate (95% CI)	0 (Ref.)	−4.45 (−6.74 to −1.12)	0.008*	0 (Ref.)	−4.84 (−5.90 to −1.25)	0.004*
Multivariate-adjusted estimate (95% CI)	0 (Ref.)	−4.20 (−6.10 to −0.94)	0.011*	0 (Ref.)	−4.64 (−5.70 to −1.05)	0.004*

TICU, transplantation intensive care medicine; CUSP, comprehensive unit-based safety program; VFDs, ventilator-free days; ICU, intensive care medicine; VAP, ventilator associated-pneumonia; IQR, interquartile range; SD, standard deviation; LOS, length of stay; CI, confidence intervals; OR, odds ratio.^*^P < 0.05.

### Secondary endpoints

After propensity score matching, in TICU settings, the wound infection was 3.4% in patients in the no CUSP group and 0.9% in patients in the CUSP group (*P* = 0.048). The duration of mechanical ventilation was 3.5 (1.3–4.3) days in patients in the no CUSP group and 3.3 (1.0–3.6) days in patients in the CUSP group (*P* = 0.001). The VFDs at day 28 were 24.5 (23.7–26.7) and 24.7 (24.3–26.9) days in patients in no CUSP and CUSP groups, respectively (*P* = 0.001). The time until VAP was 5.9 ± 1.1 and 8.6 ± 1.1 days in patients in no CUSP and CUSP groups, respectively (*P* = 0.003), and durations of antibiotic use for VAP was 18.4 ± 3.2 and 14.4 ± 2.3 days in patients in no CUSP and CUSP groups, respectively (*P* = 0.013). The ICU LOS was 5.5 (4.3–7.3) days in patients in no CUSP group and 4.5 (4.0–6.5) days in patients in the CUSP group (*P* = 0.012), and hospital LOS was 24 (17–31) and 20 (16–25) days in patients in no CUSP and CUSP groups, respectively (*P* = 0.001; Table 3). After propensity score matching in SICU, the patients in no CUSP and CUSP groups had significantly different bloodstream infection [10.1 vs. 3.0% (*P* = 0.005)], and time until VAP was 5.2 ± 0.6 days vs. 8.0 ± 0.5 days (*P* = 0.003). The duration of antibiotic use for VAP was 15.3 ± 1.5 days for patients in the no CUSP group and 10.7 ± 1.6 days for patients in the CUSP group (*P* = 0.008). The ICU LOS was 7 (3–12) days for patients in the no CUSP group and 5 (2–9.25) days for patients in the CUSP group (*P* = 0.008). The hospital LOS was 26 (16–33) days for patients in the no CUSP group and 21 (14–24) days for patients in the CUSP group (*P* = 0.008; Table 5). Before propensity score matching in TICU and SCIU, similar results were observed.

After propensity score matching in the TICU setting, multivariate logistic regression was performed. The results indicated that the patients in the CUSP group had a lower rate of wound infection (adjusted OR, 0.73; 95% CI, 0.50–0.95; *P* = 0.042; Table 4). Multivariate linear regression showed that for patients in the CUSP group, there was significant long time elapsed until VAP (adjusted estimate, +2.33 days; 95% CI, +1.25 to +3.99; *P* = 0.003) developed, and shorten durations of antibiotic use for VAP (adjusted estimate, −3.99 days; 95% CI, −7.20 to −0.08; *P* = 0.017), less ICU LOS (adjusted estimate, −0.34 days; 95% CI, −0.92 to −0.14; *P* = 0.038), and less hospital LOS (adjusted estimate, −3.54 days; 95% CI, −5.60 to −1.13; *P* = 0.003; Table 4) was observed. After propensity score matching in the SICU setting, a multivariate logistic regression analysis revealed that CUSP intervention was associated with a significant decrease (adjusted OR, 0.32; 95% CI, 0.24–0.96; *P* = 0.020) in bloodstream infection (68%; Table 6). Multivariate linear regression revealed that CUSP intervention significantly prolonged (2.80 days; 95% CI = +2.25 to +3.35) time until VAP developed, and shorten the duration of antibiotic use for VAP treatment (3.59 days; 95% CI = −6.34 to −2.84), and shorten (1.95 days; 95% CI = −2.34 to −1.04) the ICU LOS, and shorten (4.64 days; 95% CI = −5.70 to −1.05) the hospital LOS (Table 6). Similar results were observed before propensity score matching in TICU and SCIU settings.

Additionally, we compared the score of the safety management concept between no CUSP and CUSP groups. The results revealed a significant difference between the two groups (no CUSP group vs. CUSP group) in a total score for patient safety culture (149.40 ± 11.74 vs. 153.37 ± 9.74; *P* = 0.002), the feedback and communication of errors were 11.07 ± 1.62 vs. 11.84 ± 1.21 (*P* = 0.003), the degree of communication openness was 9.73 ± 1.73 vs. 10.86 ± 1.68 (*P* = 0.001), the collaboration between hospital departments was 13.44 ± 2.39 vs. 14.21 ± 2.16 (*P* = 0.011), and overall level of department safety was 3.64 ± 0.65 vs. 3.93 ± 0.61 (*P* = 0.005; Table 7).

**Table 7:** Comparison of the safety management concept scores before and after the implementation of CUSP.

**Variables**	**No CUSP (*n* = 73)**	**CUSP (*n* = 80)**	***t***	***P*-value**
Total score for patient safety culture, mean (SD)	149.40 ± 11.74	153.37 ± 9.74	−3.296	0.002*
Organize learning and continuous improvement, mean (SD)	12.19 ± 0.95	12.38 ± 1.16	−1.038	0.303
Team work within the department, mean (SD)	16.86 ± 1.94	17.05 ± 1.57	−0.716	0.476
Management's willingness and actions to promote patient safety, mean (SD)	15.36 ± 1.72	15.14 ± 1.58	0.896	0.373
Feedback and communication of errors, mean (SD)	11.07 ± 1.62	11.84 ± 1.21	−3.048	0.003*
A comprehensive understanding of patient safety, mean (SD)	14.82 ± 2.04	14.93 ± 1.69	−0.367	0.715
Hospital management support, mean (SD)	10.97 ± 1.72	11.11 ± 1.37	−0.587	0.559
A non-punitive response to a mistake, mean (SD)	9.97 ± 1.74	9.86 ± 1.88	0.387	0.700
Degree of communication openness, mean (SD)	9.73 ± 1.73	10.86 ± 1.68	−4.647	0.001*
Collaboration between hospital departments, mean (SD)	13.44 ± 2.39	14.21 ± 2.16	−2.619	0.011*
Hospital shift and transfer procedures, mean (SD)	13.41 ± 1.99	13.56 ± 2.03	−0.527	0.600
Personnel allocation, mean (SD)	12.55 ± 2.53	13.11 ± 2.48	−1.537	0.129
Frequency of adverse event reporting, mean (SD)	9.03 ± 1.98	9.32 ± 1.93	−1.005	0.318
Overall level of department safety, mean (SD)	3.64 ± 0.65	3.93 ± 0.61	−2.922	0.005*
Number of incidents reported in the past 12 months, mean (SD)	1.60 ± 0.89	1.45 ± 0.80	1.075	0.286

CUSP, comprehensive unit-based safety program. ^*^P < 0.05.

### *Post-hoc* sensitivity analysis

A sensitivity analysis was conducted based on patients requiring mechanical ventilation for ≥48 h and 72 h to assess the robustness of primary and secondary outcomes. For the primary outcome, between the no CUSP and CUSP groups, a significant difference of 29.4 vs. 9.2% (*P* = 0.025) was observed based on patients requiring mechanical ventilation for ≥48 h. The primary outcome was 31.5 vs. 11.6% (*P* = 0.037) based on patients requiring mechanical ventilation for ≥72 h in the TICU setting. In the SICU setting, a significant difference between no CUSP and CUSP groups (18.2 vs. 5.3%, *P* = 0.001) was observed based on patients requiring mechanical ventilation for ≥48 h and 18.7 vs. 4.1% (*P* = 0.001) based on patients requiring mechanical ventilation for ≥72 h. Similar results were observed for the secondary outcome for *post-hoc* sensitivity analysis in TICU and SICU was conducted for hospital infection, time until VAP developed, durations of antibiotic use for VAP treatment, ICU, and hospital LOS.

## Discussion

In this study, an uncontrolled before-and-after trial was conducted in two ICU settings (TICU and SICU). Prior to propensity score matching, the results revealed that the implementation of CSUP in patients receiving mechanical ventilation was associated with a lower incidence of VAP events and hospital-acquired infection, the longer time elapsed until the first diagnosis of VAP, fewer days of antibiotic use for VAP treatment, shorter ICU and hospital LOS, and higher awareness of safety culture. Additionally, 1:1 propensity-matched analysis also showed similar results to control confounding factors. Notably, *post-hoc* sensitivity analysis revealed that these results were generally consistent, regardless of patients requiring mechanical ventilation for ≥48 and 72 h.

Since 2020, many changes have been observed due coronavirus disease 2019 pandemic along with the return of VAP incidence, which has always been the primary concern faced by ICUs worldwide (20). High incidences of VAP are reported, despite the availability of modern preventative measures. Using bronchoscopy diagnosis, the incidences of VAP were reported to be >40/1,000 VAP ventilator-days, which was attributed to poor medical practices, guidelines, and safety culture (21). In our study, the total VAP incidence was 35.1 VAP/1,000 ventilator days in the TICU setting and 19.1 VAP/1,000 ventilator days in SIUC settings before implementing CUSP. VAP remains one of the major and most pressing public health concerns worldwide, and new strategies should be devised to prevent the incidences of VAP. CUSP is a unit structure based on its own characteristic safety culture. In CUSP, patient safety is the main priority. It integrates the science of safety into existing guidelines and norms and promotes communication, teamwork, and leadership. This enhances the implementation of guidelines and safety cultures relevant to patients, reducing the incidence of culturally related clinical events (such as CLABSI, surgical complications, and medication errors) (13–17). Thus, we hypothesized that implementing CUSP could reduce the factors interfering with the implementation and execution of the VAP bundle, thus reducing the incidence of VAP and mechanical ventilator-associated events. Currently, the concept of CUSP is predominately used by the developed countries of Europe and North America. Hence, we introduced CUSP for the first time to investigate the efficacy of CUSP in VAP prevention in China by collaborating with Johns Hopkins Armstrong Institute.

Several studies report using CUSP optimization management in clinical trials (15–18). However, our study differs from previous studies regarding the content and methodology used for analysis. First, our study focuses on the efficacy of CUSP on VAP prevention in two ICU settings, constituting a mixed set of patients. This aids in increasing the sample size, improved power of statistical analysis, and the proposed CUSP generalizability. Second, the implantation of the VAP bundle guided by CUSP comprises evidence-based strategies that provide maximum benefits, reduce barriers during the implementation, and help enhance intervention and compliance. Finally, our study has several methodological strengths over previous studies. Joinpoint regression analysis were used to identify significant shifts and trends in VAP incidence, thus determining if CUSP was a significant joinpoint. 1:1 propensity-matched analysis was used to reduce the inter-group imbalances in baseline characteristics, thereby making the groups comparable. *Post-hoc* sensitivity analysis was used to explore the stability and robustness of CUSP intervention.

A recent study reported that implementing a multipronged program such as CUSP in a single-center ICU setting could enhance the care and outcomes in patients requiring mechanical ventilation in Saudi Arabia, consistent with our results (22). However, these results show a slight decrease in the incidence of VAP, which may be due to the lack of optimized evidence-based strategies combined into the VAP bundle. Additionally, the study conducted in Saudi Arabia was a cohort study and not a randomized controlled trial. Further, the study failed to report that baseline data; hence, it is impossible to determine if the baseline data was balanced. If the baseline data was not balanced, it could likely affect the results and should be reconsidered. Our results show that the baseline characteristics (such as spontaneous bacterial peritonitis, PCT, pretransplant infection, and sex) between the two groups were asymmetric in both the TICU and SICU settings. Some studies suggest high PCT levels significantly correlated with increased incidences of VAP (23). To negate the differences in the patient for the non-random assignment, a propensity-matched analysis was performed. Before and after propensity score matching after CUSP implementation showed a significant reduction in VAP incidences in TICU and SICU settings in patients receiving mechanical ventilation. Importantly, joinpoint regression analysis demonstrated that the CUSP intervention was a significant joinpoint for altering the incidences of VAP. To sum up, the implementation of CSUP in patients requiring mechanical ventilation reduces the incidence of VAP.

Regarding the secondary outcomes, our results demonstrated that implementing CUSP led to relative risk reduction for hospital-acquired infection (wound and bloodstream infection) in our patients, which is consistent with previous studies (16, 17). This indicates that the CUSP could largely prevent healthcare-associated infection (HAI) and should be implemented in other scenarios. Further, the additional effect of CUSP interference on mechanical ventilator-associated events was evaluated. The results show that implementing CUSP can prolong the time until the VAP occurrence, shorten the duration of antibiotic use, and shorten ICU and hospital LOS. Therefore, other departments could consider implementing CUSP based on their requirements by promoting evidence-based strategies to improve the quality of patient care and safety.

To evaluate whether the efficacy of administration of CUSP was modified by inclusion criteria, such as duration of mechanical ventilation, a *post-hoc* sensitivity analysis was performed based on patients requiring mechanical ventilation for ≥48 and 72 h. The results uncovered that the implementation of CUSP still could significantly reduce the incidences of VAP, shorten the ICU and hospital LOS, etc. These results suggest that CUSP is a robust strategy for reducing the incidence of VAP.

In our study, the safety culture results indicated that the scores of total patient safety culture, three dimensions, and one individual entry in the CUSP group were significantly higher compared to the no CUSP group. This indicates that CUSP training significantly improved the attitude of medical staff toward safety, specifically in the ICU setting to ensure the safety of patients. On the contrary, no significant improvement in nine dimensions and one individual entry was observed between the two groups. In the future, while implementing CUSP, it would be beneficial to identify the defects and correct the problems for CUSP to further enhance the safety culture. The optimized CUSP can further reduce VAP incidence and other medical safety-related events by improving the patient safety culture. It is worth mentioning that *Acinetobacter baumannii* was the most common microorganism causing VAP, despite implementing CUSP in both TICU and SICU settings, thus preventing *Acinetobacter baumannii* infections might be a key step to preventing the occurrence of VAP. Hence, we should add evidence-based interventions for *Acinetobacter baumannii* (such as cleaning and disinfecting the surrounding along with proper use of antibiotics) into the VAP bundle. Therefore, CUSP leads to better implementation of the VAP bundle to achieve the desired goals in clinical settings.

Despite promising results, our study has a few limitations. First, even though implementing CUSP demonstrated impressive performance, it is not suitable for general use until it has been validated against external datasets (such as other top grade 3 and first-class hospitals in China) with large sample sizes in the prospective cohorts. Secondly, our study was retrospective, so the propensity matching analysis may not account for unobserved confounders. One of the undetected confounders could be the fitness of the patient. The patients fit enough to complete the CUSP could be inherently different from those who could not complete the study. Further, a randomized controlled trial should be performed to assess the efficacy of CUSP for VAP prevention in the future. Third, we report VAP incidences per 1,000 ventilator days, and any intervention that shortens the duration of a patient's ventilation may paradoxically raise VAP incidences, thereby underestimating the effect of the intervention on VAP outcomes. Fourth, during data collection and analysis, all known potential risk variables for VAP were not consistently extracted for every patient from the monitoring forms; hence they were not considered in multivariable analyses, especially in the SICU setting.

## Conclusion

In conclusion, the implementation of CUSP was successful, and its effect on patients requiring mechanical ventilation was evaluated. The results revealed that CUSP implementation could significantly reduce VAP incidence and nosocomial infection, prolong the time until the VAP occurrence, shorten the duration of antibiotic use, shorten ICU and hospital LOS, and improve awareness of safety culture. Other departments and hospitals should consider implementing CUSP tailored to their needs to enhance quality and patient safety.

## Data availability statement

The original contributions presented in the study are included in the article/Supplementary material, further inquiries can be directed to the corresponding authors.

## Ethics statement

All procedures performed in studies involving human participants were in accordance with the ethical standards of the institutional and/or national research committee and with the 1964 Helsinki Declaration and its later amendments or comparable ethical standards. The study was reviewed and approved by the Ethics Committee of the Third Affiliated Hospital of Sun Yat-sen University. As it was a retrospective study, informed consent was waived.

## Author contributions

XY, HY, YY, and HL conceived, designed the study, reviewed, and revised the manuscript. XY, HL, and XW drafted the manuscript. XY, MZ, YM, and JZ analyzed and interpreted all the data. XY, HY, XS, and YA prepared the figures and tables for the manuscript. All authors have read and approved the manuscript for publication.

## Supplementary material

Supplementary Table S1 Microbiological documentation of the 58 VAP occurrences in the TICU setting.

Supplementary Table S2 Microbiological documentation of the 64 VAP occurrences in the SICU setting.

Supplementary Table S3 Sensitivity analysis for primary and secondary outcomes based on patients who received mechanical ventilation for >48 and 72 h in the TICU setting.

Supplementary Table S4 Sensitivity analysis for primary and secondary outcomes based on patients who received mechanical ventilation for >48 and 72 h in the TICU setting.
