# Supplementary material for: Efficacy of comprehensive unit-based safety program to prevent ventilator associated-pneumonia for mechanically ventilated patients in China: A propensity-matched analysis
Source: Front Public Health. 2022 Dec 15;10:1029260. doi: 10.3389/fpubh.2022.1029260 (PMC9797967; doi:10.3389/fpubh.2022.1029260)
Supplement: Supplementary Table S1 — Microbiological documentation of the 58 VAP occurrences in the TICU setting. [file Table_1.docx]

**Table S1. Microbiologic documentation of the 58 VAP occurrences in the TICU setting.**

| **Variables** | **No CUSP (n=46)** | **CUSP (n=12)** |
| --- | --- | --- |
| **Candida albicans, No. (%)** | 1(2.2) | 0(0) |
| **Acinetobacter baumannii, No. (%)** | 20(43.5) | 5(41.7) |
| **Escherichia coli, No. (%)** | 1(2.2) | 0(0) |
| **Klebsiella pneumoniae, No. (%)** | 7(15.2) | 4(33.3) |
| **Elizabethkingia meningoseptica, No. (%)** | 1(2.2) | 0(0) |
| **Etenotrophomonas maltophilia, No. (%)** | 7(15.2) | 1(8.3) |
| **Pseudomonas aeruginosa, No. (%)** | 4(8.7) | 0(0) |
| **Aspergillus fumigatus, No. (%)** | 3(6.5) | 1(8.3) |
| **Burkholderia cepacia, No. (%)** | 2(4.3) | 1(8.3) |
| **MDR pathogens incidences** | 24(52.2) | 6(50) |

Abbreviations: CUSP=comprehensive unit-based safety program, TICU =transplantation intensive care medicine; MDR=multidrug resistance.
