# Supplementary material for: Efficacy of comprehensive unit-based safety program to prevent ventilator associated-pneumonia for mechanically ventilated patients in China: A propensity-matched analysis
Source: Front Public Health. 2022 Dec 15;10:1029260. doi: 10.3389/fpubh.2022.1029260 (PMC9797967; doi:10.3389/fpubh.2022.1029260)
Supplement: Supplementary Table S2 — Microbiological documentation of the 64 VAP occurrences in the SICU setting. [file Table_2.docx]

**Table S2. Microbiologic documentation of the 64 VAP occurrences in the SICU setting.**

| **Variables** | **No CUSP (n=54)** | **CUSP (n=10)** |
| --- | --- | --- |
| **Candida albicans, No. (%)** | 4(7.4) | 0(0) |
| **Acinetobacter baumannii, No. (%)** | 28(51.9) | 6(60) |
| **Burkholderia pyrrocinia, No. (%)** | 1(1.9) | 0(0) |
| **Klebsiella oxytoca, No. (%)** | 1(1.9) | 0(0) |
| **Klebsiella pneumoniae, No. (%)** | 5(9.3) | 0(0) |
| **Candida krusei, No. (%)** | 2(3.7) | 0(0) |
| **Aspergillus, No. (%)** | 1(1.9) | 0(0) |
| **Staphylococcus haemolyticus, No. (%)** | 5(9.3) | 0(0) |
| **Pseudomonas aeruginosa, No. (%)** | 7(13.0) | 2(20) |
| **Enterobacter cloacae, No. (%)** | 0(0) | 2(20) |
| **MDR pathogens incidences** | 55.6(52.2) | 5(50) |

Abbreviations: CUSP=comprehensive unit-based safety program, SICU =surgical intensive care medicine.
