# Supplementary material for: Efficacy of comprehensive unit-based safety program to prevent ventilator associated-pneumonia for mechanically ventilated patients in China: A propensity-matched analysis
Source: Front Public Health. 2022 Dec 15;10:1029260. doi: 10.3389/fpubh.2022.1029260 (PMC9797967; doi:10.3389/fpubh.2022.1029260)
Supplement: Supplementary Table S3 — Sensitivity analysis for primary and secondary outcomes based on patients who received mechanical ventilation for >48 and 72 h in the TICU setting. [file Table_3.docx]

**Table S3. Sensitivity analyses for primary and secondary outcomes based on patients who received mechanical ventilation for>48 h and 72h in the TICU setting**

| **Variables** | **Mechanical ventilation for>48 h** | | | **Mechanical ventilation for>72 h** | | |
| --- | --- | --- | --- | --- | --- | --- |
|  | **No CUSP (n=182)** | **CUSP (n=109)** | ***P* value** | **No CUSP (n=93)** | **CUSP (n=62)** | ***P* value** |
| **Primary outcome** |  |  |  |  |  |  |
| **VAP (per 1000 ventilator-days),**  **No. (‰)** | 23(29.4) | 5(9.2) | 0.025* | 18(31.5) | 5(11.6) | 0.037* |
| **Secondary outcomes** |  | 3.0(2.3-4.4) |  |  |  |  |
| **Days of mechanical ventilation**  **median (IQR),d** | 3.3(2.5-4.7) | 3.0(2.3-4.4) | 0.160 | 4.6(3.5-7.6) | 4.3(3.5-6.9) | 0.562 |
| **Total ventilator days, d** | 782 | 546.3 |  | 571.5 | 429.3 |  |
| **VFDs at day 28, median (IQR),d** | 24.7(23.3-25.5) | 25.0(23.6-25.7) | 0.160 | 23.4(20.4-24.5) | 23.7(21.1-24.5) | 0.562 |
| **Time until VAP, mean (SD), d** | 6.1(1.5) | 8.4(1.3) | 0.010* | 6.0(1.2) | 8.4(1.3) | 0.009* |
| **Reintubation, No. (%)** | 24(13.2) | 16(14.7) | 0.721 | 20(21.5) | 14(22.6) | 0.874 |
| **Pulmonary infection, No. (%)** | 59(32.4) | 21(19.4) | 0.030* | 44(47.3) | 14(22.6) | 0.042* |
| **Pleura effusion, No. (%)** | 91(50.0) | 43(39.4) | 0.168 | 56(60.2) | 27(43.5) | 0.096 |
| **Time until pleura effusion,**  **median (IQR), d** | 1(0-3.5) | 1(0-1) | 0.001* | 1(0-4) | 1(0-1) | 0.085 |
| **Wound infection, No. (%)** | 12(6.5) | 1(0.9) | 0.011* | 7(4.2) | 0(0) | 0.028* |
| **Opportunistic infection, No. (%)** | 84(46.2) | 34(31.5) | 0.016* | 53(55.2) | 24(38.7) | 0.045* |
| **Time until opportunistic infection,**  **median (IQR), d** | 0(0-10) | 0(0-4) | 0.114 | 1(0-10) | 0(0-3) | 0.060 |
| **Days of antibiotic use for VAP,**  **mean (SD), d** | 18.0(2.0) | 15.0(2.4) | 0.045* | 18.7(1.8) | 15(2.4) | 0.041* |
| **ICU LOS, median (IQR), d** | 5.6(3.5-9.3) | 4.5(2.3-6.8) | 0.037* | 8.8(4.9-15.2) | 6.4(4.4-10.6) | 0.046* |
| **Hospital LOS, median (IQR), d** | 26(18-37) | 21(17.7-29) | 0.016* | 30(20-40.8) | 25(17.7-31) | 0.020* |
| **ICU mortality, No. (%)** | 11(6.0) | 5(4.6) | 0.583 | 6(6.5) | 3(4.8) | 0.659 |
| **Hospital mortality, No. (%)** | 21(11.4) | 11(10.0) | 0.718 | 12(12.5) | 7(11.3) | 0.819 |

Abbreviations: TICU =transplantation intensive care medicine, VFDs=Ventilator-free days, ICU =intensive care medicine, VAP=ventilator associated-pneumonia, IQR=interquartile range, SD=standard deviation, LOS=length of stay. ****P*** < 0.05.
