# Supplementary material for: Efficacy of comprehensive unit-based safety program to prevent ventilator associated-pneumonia for mechanically ventilated patients in China: A propensity-matched analysis
Source: Front Public Health. 2022 Dec 15;10:1029260. doi: 10.3389/fpubh.2022.1029260 (PMC9797967; doi:10.3389/fpubh.2022.1029260)
Supplement: Supplementary Table S4 — Sensitivity analysis for primary and secondary outcomes based on patients who received mechanical ventilation for >48 and 72 h in the TICU setting. [file Table_4.docx]

**Table S4. Sensitivity analyses for primary and secondary outcomes based on patients who received mechanical ventilation for>48 h and 72h in the SICU setting**

| Variables | Mechanical ventilation for>48 h | | | Mechanical ventilation for>72 h | | |
| --- | --- | --- | --- | --- | --- | --- |
|  | **No CUSP (n=371)** | **CUSP (n=241)** | ***P* value** | **No CUSP (n=259)** | **CUSP (n=175)** | ***P* value** |
| Primary outcome |  |  |  |  |  |  |
| VAP (per 1000 ventilator-days),  No. (‰) | 45(18.2) | 10(5.3) | 0.001* | 41(18.7) | 7(4.1) | 0.001* |
| Secondary outcomes |  |  |  |  |  |  |
| Days of mechanical ventilation  median (IQR),d | 4.3(2.8-7.0) | 4.7(2.9-8.5) | 0.129 | 5.6(4.0-9.3) | 6.6(4.3-10.3) | 0.141 |
| Total ventilator days, d | 2471.3 | 1881.7 |  | 2193.6 | 1717.2 |  |
| Time until VAP, mean (SD), d | 5.2(0.9) | 8.1(0.5) | 0.008* | 5.4(0.8) | 8.1(0.5) | 0.001* |
| Bloodstream infection, No. (%) | 35(9.4) | 12(5.0) | 0.035* | 26(10.0) | 10(5.7) | 0.032* |
| Days of antibiotic use for VAP,  mean (SD), d | 15.0(1.5) | 10.9(1.4) | 0.001* | 15.1(1.6) | 11.4(1.3) | 0.001* |
| ICU LOS, median (IQR), d | 7.0(3.5-13.0) | 5.0(2.0-10.5) | 0.018* | 9(6-15) | 8.5(4.5-14) | 0.031* |
| Hospital LOS, median (IQR), d | 27(21-36) | 22(16-28) | 0.002* | 29(22-40) | 23(18-31) | 0.003* |

Abbreviations: SICU =surgical intensive care medicine, VFDs=Ventilator-free days, ICU =intensive care medicine, VAP=ventilator associated-pneumonia, IQR=interquartile range, SD=standard deviation, LOS=length of stay. ****P*** < 0.05.
